# SpO_2_/FiO_2_ as a predictor of high flow nasal cannula outcomes in children with acute hypoxemic respiratory failure

**DOI:** 10.1038/s41598-021-92893-7

**Published:** 2021-06-29

**Authors:** Ga Eun Kim, Sun Ha Choi, Mireu Park, Jae Hwa Jung, Myeongjee Lee, Soo Yeon Kim, Min Jung Kim, Yoon Hee Kim, Kyung Won Kim, Myung Hyun Sohn

**Affiliations:** 1 Department of Pediatrics, Severance Hospital, Institute of Allergy, Institute for Immunology and Immunological Diseases, Severance Biomedical Science Institute, Brain Korea 21 PLUS Project for Medical Science, Yonsei University College of Medicine, 50-1, Yonsei-ro, Seodaemun-gu, Seoul, 03722 South Korea; 2 Biostatistics Collaboration Unit, Department of Biomedical Systems Informatics, Yonsei University College of Medicine, Seoul, South Korea

**Keywords:** Respiratory distress syndrome, Paediatric research

## Abstract

The high-flow nasal cannula (HFNC) is a useful treatment modality for acute hypoxemic respiratory failure (AHRF) in children. We compared the ability of the oxygen saturation to fraction of inspired oxygen ratio (S/F) and arterial oxygen partial pressure to fraction of inspired oxygen ratio (P/F) to predict HFNC outcomes in children with AHRF. This study included children treated with HFNC due to AHRF from April 2013 to March 2019 at the Severance Children’s Hospital. HFNC failure was defined as the need for mechanical ventilation. Trends of S/F and P/F during HFNC were analyzed. To predict HFNC outcomes, a nomogram was constructed based on predictive factors. A total of 139 patients with arterial blood gas data were included in the S/F and P/F analyses. S/F < 230 at initiation showed high prediction accuracy for HFNC failure (area under the receiver operating characteristic curve: 0.751). Univariate analyses identified S/F < 230 at HFNC initiation and < 200 at 2 h (odds ratio [OR] 12.83, 95% CI 5.06–35.84), and hemato-oncologic disease (OR 3.79, 95% CI 1.12–12.78) as significant predictive factors of HFNC failure. The constructed nomogram had a highly predictive performance, with a concordance index of 0.765 and 0.831 for the exploratory and validation groups, respectively. S/F may be used as a predictor of HFNC outcomes. Our nomogram with S/F for HFNC failure within 2 h may prevent delayed intubation in children with AHRF.

## Introduction

High flow nasal cannula (HFNC) treatment has been described as a safe and useful technique to deliver heated and humidified oxygen to patients with acute hypoxemic respiratory failure (AHRF)^1^. The reported beneficial effects of HFNC include a decrease in physiological dead space, an improvement in oxygenation, and a reduction in dyspnea by supplying oxygen at a high flow rate. As such, it may be used as a next-step respiratory support after nasal prongs or an oxygen mask in patients with respiratory failure^2,3^. It has been reported that the administration of HFNC is associated with a reduction in the rate of intubation with mechanical ventilation (MV) in patients with AHRF^2^. On the other hand, HFNC in infants with bronchiolitis did not decrease in an intubation group compared with a standard oxygen therapy group in a recent randomized trial^4^. In any case, if respiratory symptoms, signs, or laboratory findings, including blood gas, do not improve after implementation of HFNC, a more aggressive ventilation technique, such as noninvasive^5^ or invasive MV, should be considered. Identifying which patients may respond to HFNC and who may need MV can be a challenging decision^3^. The decision to initiate MV is a critical one, as delayed intubation has proven to be a concern during HFNC treatment^3^. Therefore, predicting the outcome of HFNC at an optimal time is crucial.

To date, improvements in gas exchange and respiratory rates (RRs) have reportedly remained a predictor of successful HFNC outcomes^6,7^. In contrast, clinical parameters that warrant a subsequent need for intubation include absence of oxygenation improvement, absence of significant decrease in RR, presence of additional organ failure, or persistence of thoraco-abdominal asynchrony^7,8^. The ratio of arterial oxygen partial pressure and fraction of inspired oxygen (PaO_2_/FiO_2_; P/F) has been suggested as an outcome predictor for noninvasive ventilation in patients with AHRF^9^. However, P/F requires arterial blood gas sampling; this procedure is invasive and not readily available in clinical practice, especially for children^10^. The oxygen saturation (SpO_2_)/FiO_2_ (S/F) ratio is a noninvasive, easily detectable, and readily available parameter that may be used as a surrogate marker for P/F in children^10–12^. Furthermore, low S/F has been reported in cases of severe AHRF^13^.

The prediction of HFNC outcomes may help clinicians make a timely and optimal decision to intubate children with AHRF. Given that P/F may predict HFNC outcomes, we sought to identify whether S/F could predict HFNC outcomes as well. We also aimed to construct a nomogram as a shortcut prediction tool for HFNC outcomes.

## Methods

### Patients

A retrospective chart review of children treated with HFNC due to AHRF was conducted at the Severance Children’s Hospital, a single tertiary center, between April 2013 and March 2019. All patients who received HFNC treatment for AHRF were included in the study. Exclusion criteria were age > 18 years, indication for endotracheal intubation within 1 h of HFNC initiation, post-extubation state, and congenital heart disease^14,15^. Patients were divided into two groups: HFNC success and HFNC failure. HFNC failure was defined as the need for invasive MV due to progressive respiratory failure; the intubation decisions were based on the following criteria: a clinical deterioration such as tachypnea, chest retraction in respiratory status, a lack of improvement in signs of high respiratory muscle workload, or deterioration of the blood gas analysis, hemodynamic instability, and deterioration of neurological status^2^. HFNC success was defined as an improvement of respiratory distress with HFNC. A total of 419 children were treated using HFNC during the aforementioned period. Among these, 165 patients were excluded for the following reasons: 47 patients needed endotracheal intubation within 1 h of HFNC initiation, 52 patients were in the post-extubation state, 60 patients had congenital heart disease, and 6 patients were treated with noninvasive ventilation (NIV) due to progressive respiratory failure. Among the remaining 254 children, 139 who had available arterial blood gas data during the HFNC treatment were assigned to the exploratory group, and 114 without arterial blood gas data were included in the validation group. The Institutional Review Board of Severance Hospital approved this study (Seoul, Korea, Institutional Review Board 4-2020-0036) and granted the exemption of informed consent. Our study conformed to the Declaration of Helsinki, and all methods were performed following relevant guidelines and regulations.

### Variable measurements and definitions

Demographic data such as age, sex, weight, underlying condition, and etiology of respiratory failure were recorded. Physiologic clinical variables such as the SpO_2_, FiO_2_, RR, heart rate (HR), and the flow rate of gas delivered (L/min) were also obtained at HFNC initiation. The P/F was obtained from the arterial blood gas analysis at the time of HFNC initiation.

To estimate oxygenation, we calculated the S/F as a noninvasive alternative to the P/F^11^. The SpO_2_ and FiO_2_ were recorded at 1, 2, 4, and 12 h after HFNC initiation, and the corresponding series of S/F were calculated. HFNC initiation was defined as the point of the when HFNC treatment was started.

We evaluated the S/F as either a continuous or categorical variable, based on whether the patients achieved the therapeutic goal of S/F > 200^16^. The continuous S/F variable was substituted for the new categorical form to construct a nomogram model.

### Device description and management

HFNC was implemented using the Optiflow (Fisher & Paykel Healthcare, Auckland, New Zealand) device, which comprises an air mixing device, a heated humidifier, a heated gas humidification chamber (MR290), a high-performance breathing circuit (900PT561), and a unique wide bore nasal cannula. The HFNC settings were determined by each attending physician.

### Statistical analyses

The patients’ characteristics are summarized using numbers and percentages for categorical variables, and medians (interquartile range) for continuous variables. For intergroup comparisons, the Mann–Whitney U test was used for continuous variables, and the chi-squared test or Fisher’s exact test was used for categorical variables.

Receiver operating characteristic (ROC) curve analyses were performed to assess the S/F and P/F cutoff for HFNC outcomes. The area under the ROC curve (AUC was calculated as a measure of predictive capacity. The difference of AUC was determined using Delong’s method^17^.

Univariate logistic regression analysis was used to identify independent predictive risk factors for HFNC outcomes. Factors with a *P* value < 0.05 in the univariate analyses were included in the prediction model. The effect of each potential risk factor was denoted by the odds ratio (OR) and its 95% confidence interval (CI).

A nomogram was constructed based on selected predictive factors identified using the multivariate logistic regression model of the exploratory group data. The goodness of fit for each nomogram was verified using the Hosmer–Lemshow test. The discrimination ability of the nomogram was analyzed using the AUC. A calibration curve was generated to assess the discriminative performance and predictive accuracy of the nomogram. The proposed prediction model was verified through external validation of the independent data. Statistical analyses were performed using Statistical Package for the Social Sciences version 25 and R (version 3.6.1, The R Foundation for Statistical Computing, Vienna, Austria). A two-sided *P* value < 0.05 was considered statistically significant.

## Results

### General characteristics

A total of 139 children with AHRF were included in the exploratory cohort. Baseline characteristics of the study population are presented in Table 1. Fifty-nine (42.4%) patients who required intubation with MV were categorized as the HFNC failure group. The median time of HFNC treatment was 14.1 h (interquartile range, 4.5–17.9), and 50 (83%) patients were intubated within 24 h. The leading cause for AHRF was pneumonia, which accounted for 67% of the total cases. Other etiologies for AHRF were bronchiolitis, bronchospasm, and acute respiratory distress syndrome (ARDS). HFNC success was statistically significant in children with bronchiolitis (*P* = 0.041) and marginally significant in children with bronchospasm. However, HFNC treatment did not show any statistical significance in AHRF due to other etiologies. The most frequent underlying diseases associated with AHRF were neuromuscular disease (61.1%) and respiratory disease (12.2%); 17 children did not have any underlying disease. Patients with underlying hemato-oncologic diseases with AHRF frequently needed intubation with MV after HFNC treatment (*P* = 0.021).

**Table 1:** Patient characteristics in the exploratory group.

Characteristics	HFNC success	HFNC failure	*P*-value
	(n = 80)	(n = 59)	
Age, years	3.9 (1.2, 9.3)	6.0 (1.69, 13.7)	0.087
Male, n (%)	51 (63.7)	32 (53.3)	0.214
**Cause of respiratory failure**			
Pneumonia (n = 94)	51 (63.7)	43 (72.9)	0.255
Bronchiolitis (n = 16)	13 (16.3)	3 (5.1)	0.041
Bronchospasm (n = 9)	8 (10.0)	1 (1.7)	0.078
ARDS (n = 9)	3 (3.8)	6 (10.2)	0.169
Upper airway disease (n = 3)	1 (1.3)	2 (3.4)	0.574
**Underlying disease**			
Neuromuscular disease (n = 84)	48 (60.0)	36 (61.0)	0.904
Pulmonary disease (n = 17)	12 (15.0)	5 (8.5)	0.246
Hemato-oncology (n = 14)	4 (5)	10 (16.9)	0.021
Others (n = 7)^a^	3 (3.8)	4 (6.8)	0.419

Data are expressed as n (%) or medians (interquartile ranges).

n, numbers; HFNC, high flow nasal cannula; ARDS, acute respiratory distress syndrome; FiO_2_, fraction of inspired oxygen; SpO_2_, pulse oximetry oxygen saturation; P/F, PaO_2_/FiO_2_.

^a^Others include systemic lupus erythematosus (three patients), metabolic disorder (two patients), and chronic kidney disorder (two patients).

The validation cohort comprised 114 patients. No significant differences were found between the exploratory and validation groups. The results in the validation group were consistent with those in the exploratory group, and S/F was significantly lower in the HNFC failure group (*P* < 0.001). The patients with hemato-oncologic disease frequently needed MV in the validation group (*P* = 0.039) (Supplementary Table S1).

### Respiratory variables and serial S/F monitoring during HFNC

Table 2 shows the respiratory variables at initiation and serial S/F monitoring between the HFNC success and failure groups during HFNC treatment. The SpO_2_ at HFNC initiation was significantly lower in the HFNC failure group than in the HFNC success group (*P* < 0.001). Patients in the HFNC failure group were treated with higher FiO_2_ at initiation compared with the patients in the HFNC success group (*P* = 0.001). Signs of respiratory distress such as RR and HR at HFNC initiation did not significantly differ between the two groups.

**Table 2:** Respiratory variables and serial S/F monitoring between HFNC success and failure groups during HFNC.

	HFNC success	HFNC failure	*P*-value
	(n = 80)	(n = 59)	
Respiratory rate	35 (27.5, 42.5)	29 (24.7, 40.7)	0.424
Heart rate	152 (125.5, 163.0)	153.0 (137, 167.7)	0.077
**HFNC setting at initiation**			
FiO_2_	0.4 (0.3, 0.5)	0.45 (0.38, 0.6)	0.001
Flow/weight	1.0 (0.8, 1.3)	1.0 (0.6, 1.4)	0.503
SpO_2_ at initiation	97.0 (95.0, 99.0)	89.0 (86.2, 92.7)	< 0.001
P/F at initiation	263.6 (213.4, 340.0)	191.7 (143.5, 286.5)	0.004
**S/F**			
Initiation (n = 139)	242.5 (200.0, 320.0)	202.5 (153.3, 229.3)	< 0.001
1 h (n = 139)	243.7 (200.0, 306.4)	214.1 (161.8, 236.8)	< 0.001
2 h (n = 136)	247.5 (226.2, 323.3)	196.0 (153.9, 246.2)	< 0.001
4 h (n = 126)	250.0 (283.1, 326.7)	221.1 (168.5, 270.7)	< 0.001
12 h (n = 102)	250.0 (212.4, 330.0)	212.4 (146.4, 245.6)	< 0.001

Data are expressed as n (%) or medians (interquartile ranges).

n, numbers; HFNC, high flow nasal cannula; FiO_2_, fraction of inspired oxygen; SpO_2_, pulse oximetry oxygen saturation; P/F, PaO_2_/FiO_2_; S/F, SpO_2_/FiO_2_.

The P/F at initiation in the HFNC failure group was significantly lower than that in the HFNC success group (*P* = 0.004). We confirmed that S/F was positively correlated with P/F, which showed a linear relationship using the regression equation (S/F at initiation = 135.199 + 0.375 × P/F at initiation, *P* < 0.001, Supplementary Figure S1). Therefore, S/F was recorded as a respiratory oxygenation variable through serial monitoring. The serial S/F displayed significant differences between the groups during HFNC treatment (*P* < 0.001). The mean S/F profile plot over time based on a linear mixed model is shown in Supplementary Figure S2. The S/F of patients in the HFNC success group improved consistently during the initial 12 h after HFNC treatment. In the HFNC failure group, the S/F fluctuated within the first 4 h after HFNC initiation, with a minimum value of 197.72 (185.4–209.9) at 2 h. However, when analyzing the difference in the S/F ratio over time, the amount of change in the S/F ratio was not a meaningful variable as a predictor of HFNC failure (Supplementary Table S2).

**Figure 1:**
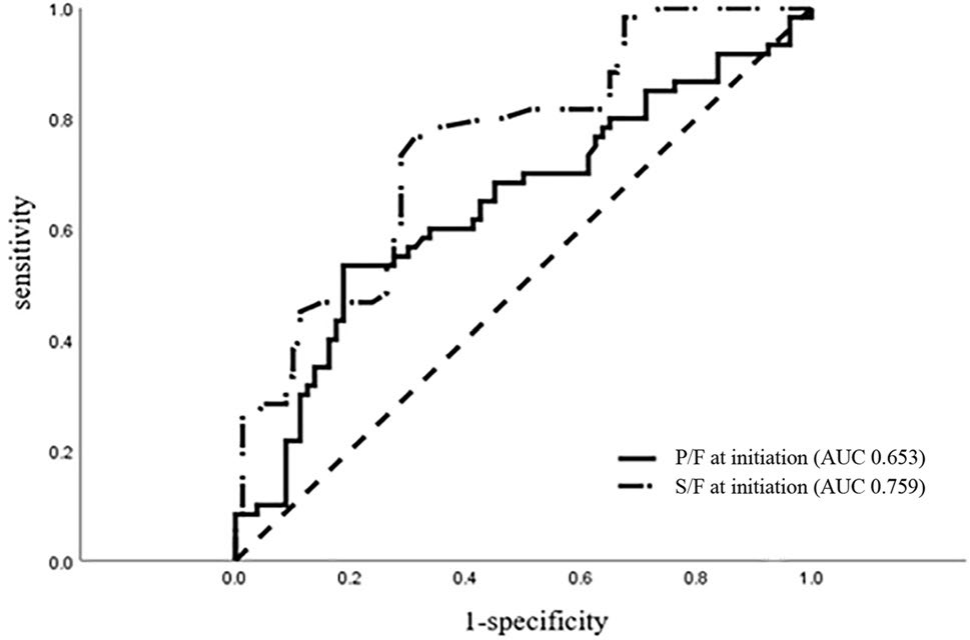
Comparison of receiver operating characteristic curve of P/F and S/F for predicting HFNC failure. The AUC was 0.653 for P/F at initiation and 0.759 for S/F at initiation. The difference between the AUCs was statistically significant (*P* = 0.005 by Delong’s method). P/F, ratio of arterial oxygen partial pressure to fraction of inspired oxygen (PaO_2_/FiO_2_); S/F, ratio of oxygen saturation to fraction of inspired oxygen (SpO_2_)/FiO_2_; AUC, area under the curve; HFNC, high flow nasal cannula.

The AUC of S/F at initiation for predicting HFNC failure was 0.759, and the optimal cutoff was 230 (Fig. 1). The S/F < 230 showed 78.0% sensitivity and 68.7% specificity. The AUC for P/F at HNFC initiation was 0.643, and a cutoff < 195 showed 54.2% sensitivity and 81.2% specificity for predicting HFNC failure. The prediction power of S/F was observed to be better than that of P/F (*P* = 0.005).

### Univariate logistic regression analysis of predictor of HFNC

Predictive potential risk factors for HFNC failure were identified using univariate logistic regression analyses in the exploratory group. The following variables were included in the analysis: RR, HR, flow/weight of HFNC setting at initiation, underlying disease, and newly categorized variables using an S/F < 230 at HNFC initiation and < 200 at 2 h (Table 3), which was identified based on the result of an analysis using various S/F cutoffs (Supplementary Table S3). A combination of S/F < 230 at HNFC initiation and S/F < 200 at 2 h (OR 13.067; 95% CI 5.06–35.84, *P* < 0.001), and hemato-oncologic disease (OR 3.799; 95% CI 1.129–12.78, *P* = 0.031) were significantly associated with HFNC failure. Therefore, we chose these two variables (combination of S/F < 230 at initiation and < 200 at 2 h), and the presence of hemato-oncologic disease for multiple logistic regression analysis to construct a nomogram.

**Table 3:** Univariate analysis of predictive factors for HFNC failure.

	Odds ratio	95% CI	*P*-value
Respiratory rate	0.988	0.959–1.018	0.424
Heart rate	1.013	0.998–1.026	0.055
Flow/weight of HFNC setting	1.055	0.597–1.862	0.8541
**Achievement of therapeutic goal**^a^ **of S/F**			
S/F at initiation ≥ 230 and S/F at 2 h < 200	1 [ref]		
S/F at initiation < 230 and S/F at 2 h ≥ 200	3.967	1.286–8.136	0.002
S/F at initiation < 230 and S/F at 2 h < 200	13.067	5.06–35.84	< 0.001
**Underlying disease**			
Neuromuscular disease	1.072	0.540–2.130	0.841
Pulmonary disease	0.515	0.171–1.551	0.384
Hemato-oncology	3.799	1.129–12.78	0.031

Data are expressed as odds ratios with 95% confidence intervals.

HFNC, high flow nasal cannula; S/F: SpO_2_/FiO_2_.

^a^Therapeutic goal: S/F ≥ 200 after initiation of HFNC.

### Nomogram construction and validation with HFNC

**Figure 2:**
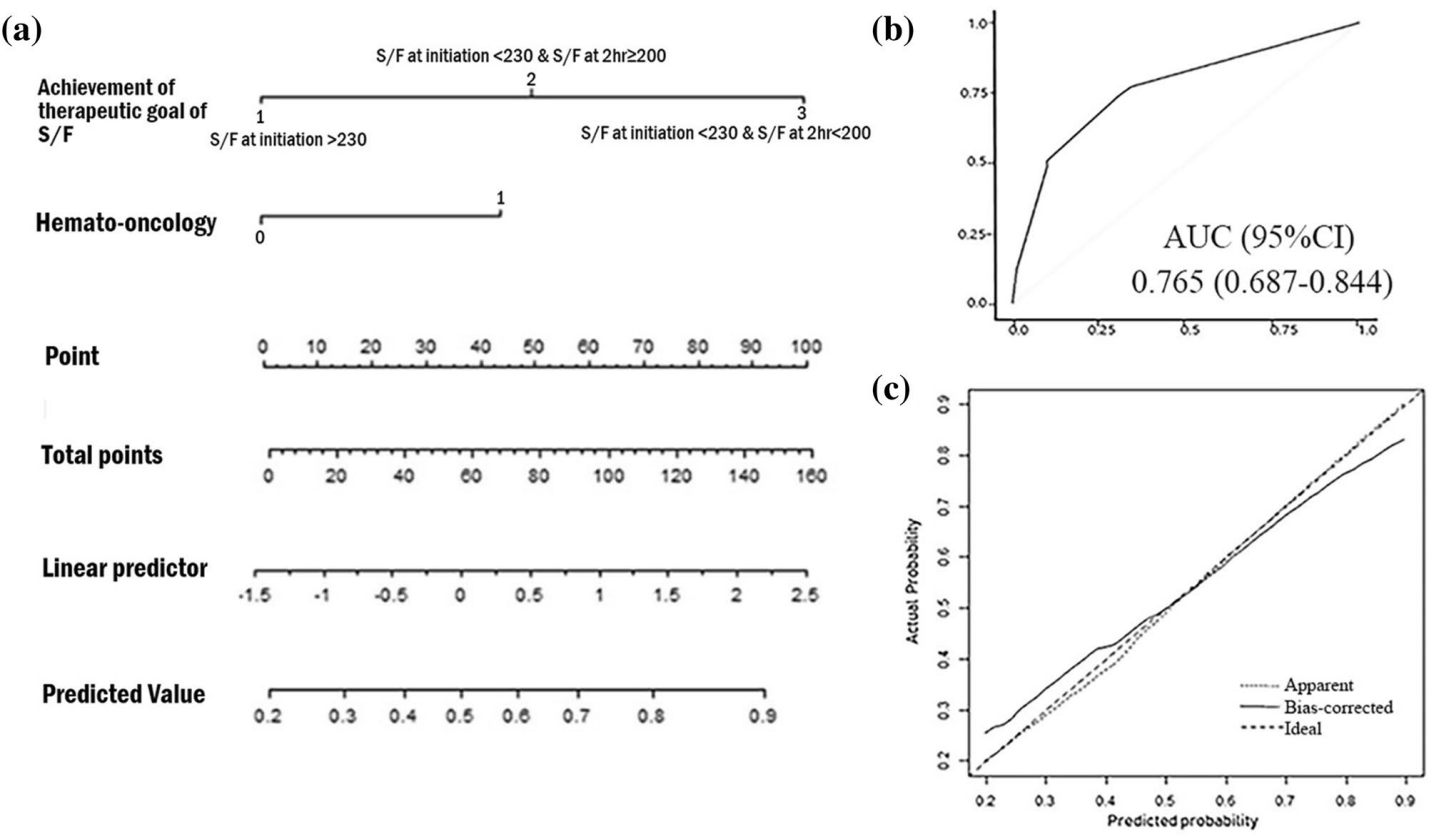
Constructed nomogram and performance of the model in the training cohort for predicting HFNC outcomes. (**a**) Nomogram according to clinical indices for predicting HFNC outcomes. The nomogram is used by adding up points identified on the points scale for each variable. The points of the three predictors should be added to calculate the total points. The straight edge should be aligned to the “total points,” and the predicted value would be visible on the last line. (**b**) ROC curve of the nomogram in predicting HFNC failure in the training cohort. AUC shows the ability of the nomogram. (**c**) Calibration curve of nomogram in the training cohort. The x-axis is the predicted probability from the nomogram, and the y-axis is the actual probability. The dashed line represents performance of the ideal nomogram (predicted outcome perfectly corresponds with actual outcome). The dotted line represents the apparent accuracy of our nomogram without correction. The solid line represents bootstrap-corrected performance of our nomogram. AUC, area under the ROC curve; ROC, receiver operating characteristic; HFNC, high flow nasal cannula.

Figure 2a shows a nomogram that was constructed according to two independent predictors from the multiple logistic regression analysis. The Hosmer–Lemshow test showed that the fit for multiple logistic regression model was good (*P* > 0.9999). In the exploratory group, the ROC curve according to the predicted probability of the multiple logistic regression analysis is shown in Fig. 2b, and the AUC was 0.765 (95% CI, 0.687–0.844). The calibration curve showed that the model was close to ideal (Fig. 2c). A higher score calculated in the nomogram was associated with a higher likelihood of HFNC failure. For example, a patient with a hemato-oncologic disease whose S/F was 190 initially and 210 at 2 h would achieve a total score of 90, which corresponded to an approximately 70% HFNC failure risk.

**Figure 3:**
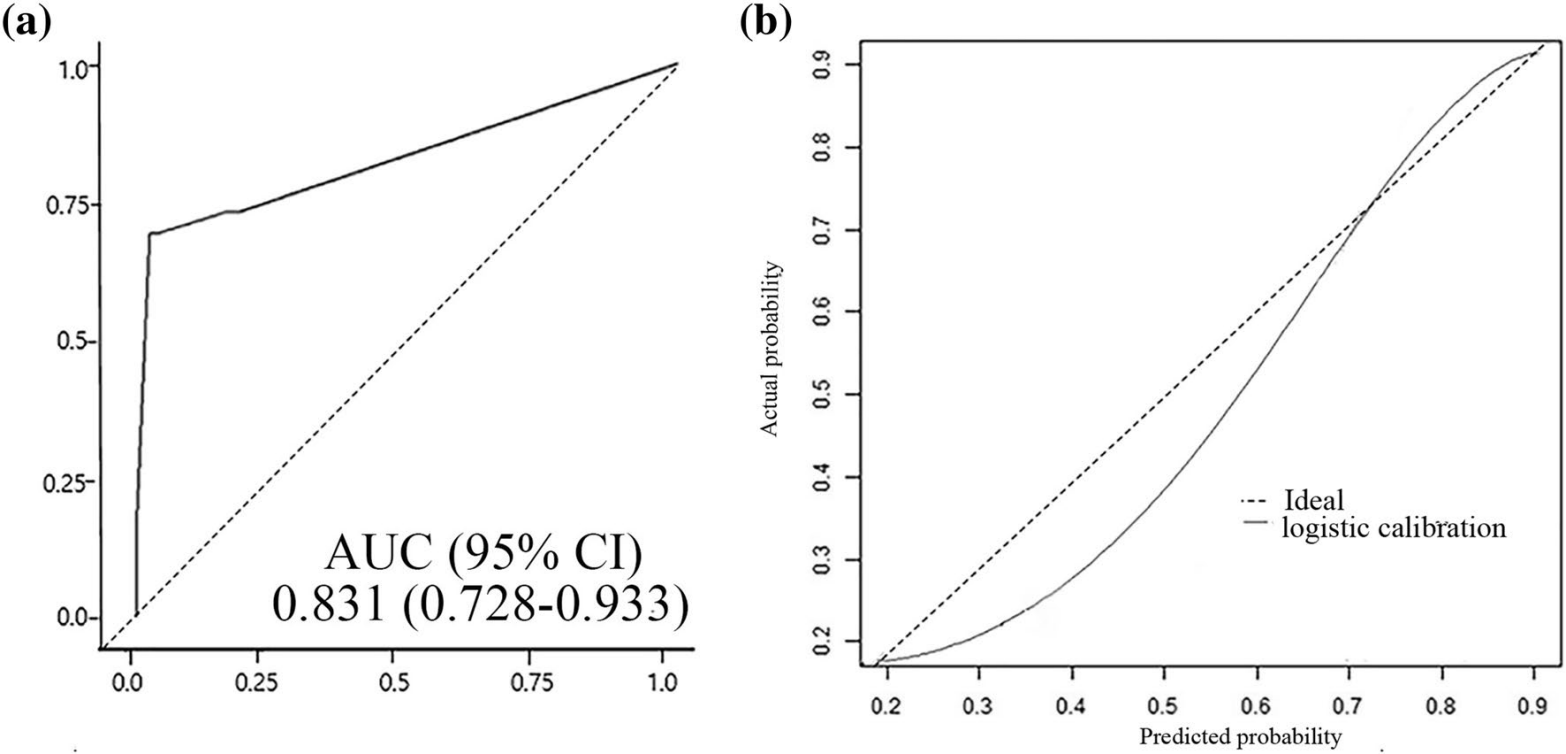
Validation of nomogram for predicting HFNC outcomes in patients with AHRF. (**a**) ROC curve of the nomogram with 114 patients in the validation cohort. (**b**) Calibration plot of the nomogram in the validation cohort. The black line indicates logistic calibration of the validation cohort. The x-axis is the predicted probability from nomogram, and the y-axis is the actual probability. The dashed line represents performance of the ideal nomogram (predicted outcome perfectly corresponds with actual outcome). ROC, receiver operating characteristic; HFNC, high flow nasal cannula; AHRF, acute hypoxemic respiratory failure.

The nomogram also displayed its accuracy in the validation group, with an AUC = 0.831 (95% CI, 0.728–0.933) (Fig. 3a). The calibration curve presented an optimal agreement between the predicted and actual probabilities in the validation group (Fig. 3b).

## Discussion

Our study showed that S/F, a noninvasive continuous monitoring variable, might be a good predictor for HFNC outcomes in children with AHRF. We created a nomogram for HFNC failure using S/F as a variable at initiation and 2 h after HFNC implementation, as well as for the presence of hemato-oncologic disease, as a shortcut prediction tool.

Multiple studies have shown that S/F has a good correlation with P/F in patients with respiratory failure^11,12^. Our study showed similarly consistent results with a good correlation between S/F and P/F. Furthermore, we showed that S/F had a better predictive power for HFNC failure compared with that of P/F. The best predictive S/F cutoff at initiation of HFNC was 230 in our study, which was higher than that in a previous study that reported S/F < 195 during the first hour of treatment to be associated with HFNC failure^18^. The aforementioned study included patients with cardiac comorbidity, while we excluded children with congenital heart diseases because they have distinct S/F levels due to their underlying diseases.

We acknowledge that our inclusion criteria might have led to different S/F cutoff levels for the prediction of HFNC failure. Fine-tuning of S/F cutoff is essential to achieve an excellent prediction power for HFNC failure. Accordingly, we used a previously reported therapeutic goal of S/F and combined it with our initial S/F cutoff to create a categorical variable^16^. Finally, an S/F cutoff of < 230 at initiation and < 200 at 2 h was observed to have a remarkable prediction power (OR, 13.067; 95% CI 5.06–35.84). An emerging issue for HFNC implementation in patients with AHRF is the concern of delayed intubation, which might worsen the clinical deterioration^19,20^. Therefore, timely and appropriate identification of HFNC failure is crucial.

Several indices such as P/F and S/F have been reported to be predictors for HFNC outcome^16,21^. The respiratory rate oxygenation (ROX) index, the ratio of SpO_2_/FiO_2_ to RR, has recently been proposed to be a better predictor for HFNC failure compared with that of S/F alone in adults^3,22^. Moreover, the heart rate, acidosis, consciousness, oxygenation, and respiratory rate (HACOR) score has been suggested as a predictive tool for noninvasive ventilation (NIV) failure in adults^23^. We also evaluated vital sign changes over time, including changes in HR and RR, although they did not differ between the HFNC success and failure groups (Supplementary Table S4). However, these indices using actual respiratory variables are difficult to apply directly in children with AHRF due to the variability of RR with age in children^24^. Therefore, our categorical S/F variable may help clinicians decide within 2 h whether the next step respiratory support, including noninvasive or invasive MV, should be performed.

Our study showed that the presence of an underlying hemato-oncologic disease was independently associated with HFNC failure, suggesting the deleterious effect of such a disease on HFNC outcome. Our findings support those of a previous study that reported that HFNC neither improved discomfort nor decreased the need for intubation in patients with hemato-oncologic diseases^25^. In our study, 70% of patients with hemato-oncologic diseases in the HFNC failure group had a severe AHRF with a P/F of 150 mmHg at HFNC initiation, and pneumonia was the cause of AHRF in all patients with hemato-oncologic diseases. This result parallels that of a previous study, which showed that the etiology of AHRF (pneumonia, OR 11.2) was a significant risk factor for HFNC failure^26^. HFNC failure in children with hemato-oncologic diseases might lead to various clinical conditions, complications, and problems unrelated to AHRF^27^. Further, the conditions associated with the hemato-oncologic diseases might not be influenced by the mode of oxygen delivery^25^. Moreover, supporting evidence has shown that the time needed to improve oxygenation during AHRF might be longer in patients with hemato-oncologic diseases than in other patients^28^. These findings may explain why the presence of underlying hemato-oncologic disease was identified as an independent parameter for HFNC failure in our study data. As such, HFNC in patients with hemato-oncologic diseases and AHRF should be monitored with more caution.

Our study is the first to build a nomogram that predicts HFNC failure in children with AHRF. With the help of our nomogram, which was constructed using a combination of time-series S/F and hemato-oncologic disease as predictors, clinicians may estimate the individual probability of HFNC outcome in a patient without the need for an invasive examination. The nomogram, based on time-series S/F at initiation and at 2 h, can guide the next respiratory support, including intubation in children with AHRF regardless of their etiologies. Furthermore, we included both internal and external validation procedures, which demonstrated strong discrimination and calibration. With the ability to estimate individual risk in an easy to use and straightforward manner, we believe that our nomogram has an advantage over simple predictors.

Our results should be interpreted with caution, as six patients who required escalation to other NIVs were not assessed. NIV was actively implemented during the middle of the study period; consequently, those patients were excluded to maintain the homogeneity of the study. We also acknowledge the inclusion of measurements in the analysis that were performed with > 97% SpO_2_, where the oxyhemoglobin dissociation curves might have been unchanged. However, some children with AHRF who receive appropriate oxygen therapy have an SpO_2_ > 97%^29^. Real-world clinical evidence in children with AHRF is necessary, and it can reasonably include patients with > 97% SpO_2_ to reflect current practice. A good correlation between S/F and P/F using data with S/F > 97% has been also demonstrated, which is consistent with our results^30^. Third, FiO_2_ based on the liter flow with the HFNC could potentially be overestimated due to entrainment of room air, especially when the HFNC flow rate is lower than the patient’s inspiratory flow rate. Therefore, we applied the flow rates^3–5,31^ from 0.5 to 2.0 L/kg/min up to a maximum of 30 L/min to prevent these problems. Fourth, this study was retrospective with inevitable limitations such as lack of data in the medical record, provider-dependent decision for intubation even though the physicians followed the institutional protocols, and uneven age distribution between the HFNC success and failure groups despite not reaching statistical significance. Lastly, we acknowledge that the validation cohort had a lower HFNC failure rate and higher S/F ratio compared with that of the exploratory cohort, even though both groups had a comparable composition of underlying disease and proportion of causative disease. Thus, the constructed nomogram needs to be validated in another independent cohort, including children with severe AHRF.

In conclusion, S/F may be an easy-to-use predictor of HFNC outcomes in children with AHRF. We constructed a nomogram using S/F for HFNC failure within 2 h, which may prevent delayed intubation in children with AHRF.

## Supplementary Information

Supplementary Information.

## Abbreviations

AHRF: Acute hypoxemic respiratory failure
ARDS: Acute respiratory distress syndrome
AUC: Area under the curve
CI: Confidence interval
HFNC: High flow nasal cannula
HR: Heart rate
MV: Mechanical ventilation
NIV: Noninvasive ventilation
OR: Odds ratio
P/F: Arterial oxygen partial pressure to fraction of inspired oxygen ratio (PaO_2_/FiO_2_)
ROC: Receiver operating characteristic
ROX: Ratio of SpO_2_/FiO_2_ to RR index
RR: Respiratory rate
S/F: Oxygen saturation to fraction of inspired oxygen ratio (SpO_2_)/FiO_2_
