# Supplementary material for: SpO2/FiO2 as a predictor of high flow nasal cannula outcomes in children with acute hypoxemic respiratory failure
Source: Sci Rep. 2021 Jun 29;11:13439. doi: 10.1038/s41598-021-92893-7 (PMC8242081; doi:10.1038/s41598-021-92893-7)
Supplement: Supplementary file 1 — Supplementary Information. [file 41598_2021_92893_MOESM1_ESM.pdf]

SpO_2/FiO_2 as a PREDICTOR of HIGH FLOW NASAL CANNULA OUTCOMES in CHILDREN WITH ACUTE HYPOXEMIC RESPIRATORY FAILURE

Ga Eun Kim[1], Sun Ha Choi[1], Mireu Park[1], Jae Hwa Jung[1], Myeongjee Lee[2], Soo Yeon Kim[1], Min Jung Kim[1], Yoon Hee Kim[1], Kyung Won Kim[1], Myung Hyun Sohn[1]

SUPPLEMENTAL MATERIAL

Supplementary Table S1. Patient characteristics in the validation group

**Characteristics**	**HFNC success (n=86)**	**HFNC failure (n=28)**	***P*-value**
**Age, years**	1.7 (0.8, 5.6)	3.4 (1.3, 13.3).	0.042
**Male, n (%)**	53.0 (61.7)	18.0 (64.2)	0.064
**RR**	32.0 (25.0, 40.0)	32.5 (24.2, 46.0)	0.919
**HR**	147.0 (121.0, 163.0)	144.5 (134.2, 168.0)	0.206
**S/F at initiation**	250.0 (237.5, 326.6)	194.0 (184.0, 246.9)	<0.001
**Cause of respiratory failure**			
**Pneumonia (n=84)**	59 (68.6)	25 (89.2)	0.031
**Bronchiolitis (n=18)**	17 (19.8)	1 (3.6)	0.041
**Bronchospasm (n=7)**	6 (6.9)	1 (3.6)	0.451
**Upper airway disease (n=5)**	4 (4.6)	1 (3.6)	0.642

**Underlying disease**			
**Neuromuscular disease (n=56)**	41 (47.4)	19 (67.8)	0.037
**Pulmonary disease (n=22)**	20 (23.2)	2 (7.1)	0.061
**Hema-oncology (n=9)**	4 (4.6)	5 (17.8)	0.039
**Others (n=2)***	0 (0)	2 (7.1)	0.059

Data are expressed as n (%) or medians (interquartile ranges).

*Others include metabolic disorder (two patients)

n, numbers; HFNC, high flow nasal cannula; ARDS, acute respiratory distress syndrome; HR, heart rate; RR, respiratory rate; S/F, oxygen saturation to fraction of inspired oxygen ratio

Supplementary Table S2. Changes of SpO_2/FiO_2 between success and failure groups

	Success group (n=82)	Failure group (n=60)	*P*-value
S/F ratio			
$\Delta SpO_2/FiO_2$ at 1 h (n=139)	0 [-3.9, 6.8]	17.2[0.00, 54.19]	0.034
$\Delta SpO_2/FiO_2$ at 2 h (n=136)	2.9 [-3.3, 8.5]	7.5[-19.3, 20.2]	0.784
$\Delta SpO_2/FiO_2$ at 4 h (n=126)	5.8 [-5.0, 47.7]	9.0[-3.6, 29.6]	0.889
$\Delta SpO_2/FiO_2$ at 12 h (n=102)	6.6 [-1.3, 79.5]	19.8[-22.2, 48.6]	0.991

Data are expressed as medians (interquartile ranges).

n, numbers; FiO_2, fraction of inspired oxygen; SpO_2, pulse oximetry oxygen saturation; S/F, SpO_2/FiO_2

Supplementary Table S3. Univariate analysis of predictive factors for HFNC failure

	Odds ratio	95% CI	*P*-value
**S/F at initiation**			
**S/F**	0.985	0.978–0.991	<0.0001
**S/F <200**	2.80	1.364–5.787	<0.0001
**S/F <230**	7.00	3.29–14.89	<0.0001
**Achievement of therapeutic goal of S/F**			
**S/F at 1 h <200**	3.032	1.449–6.346	0.003
**S/F at 2 h <200**	7.250	3.124–16.824	<0.0001

HFNC, high-flow nasal cannula; S/F, oxygen saturation to fraction of inspired oxygen ratio

Supplementary Table S4. Serial respiratory variables between HFNC success and failure groups during HFNC

	HFNC success (n=80)	HFNC failure (n=59)	*P*-value
**HR**			
**Initiation (n=139)**	242.5 (200.0,320.0)	202.5 (153.3,229.3)	<0.001
1 h (n=139)	140 (120,159)	140 (130,160)	0.471
2 h (n=133)	140 (119.7,156,7)	142 (129.0, 155.7)	0.319
4 h (n=123)	133.0 (114.0,147.0)	135.0 (119.5,150.0)	0.344
12 h (n=107)	130.0 (110.5,145.0)	139.0 (123.0,145.0)	
**RR**			
**Initiation (n=139)**	35.0 (27.5,42.5)	29.0 (24.7,40.7)	0.424
1 h (n=139)	33 (25.2,42.0)	33 (25.0,40.0)	0.661
2 h (n=133)	32.0 (28.0,40.0)	35 (30.0,44.0)	0.179
4 h (n= 123)	30.0 (26.0,36.0)	31.0 (28.0,38.5)	0.122
12 h (n=107)	32.0 (25.0,39.0)	35.0 (30.0,43.0)	0.151

Data are expressed as n (%) or medians (interquartile ranges).

n, numbers; HFNC, high flow nasal cannula; FiO_2, fraction of inspired oxygen; SpO_2, pulse oximetry oxygen saturation; P/F, PaO_2/FiO_2; S/F, SpO_2/FiO_2

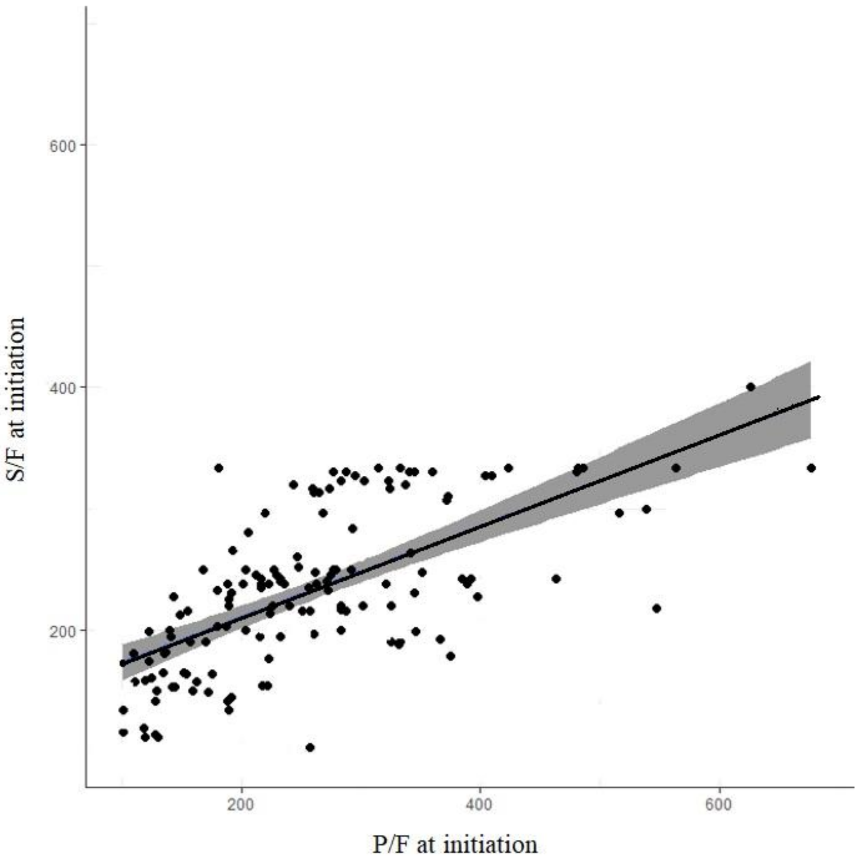

Supplementary Figure 1. Scatterplot for S/F at initiation vs P/F at initiation.

The line represents the best-fit linear relationship; S/F at initiation = 135.199 + 0.375 × P/F at initiation (P<0.001). (Correlation=0.662, 95% confidence interval: 0.557–0.746, P<0.001).

S/F, ratio of oxygen saturation and fraction of inspired oxygen (SpO_2)/FiO_2; P/F, the ratio of arterial oxygen partial pressure on fraction of inspired oxygen (PaO_2/FiO_2)

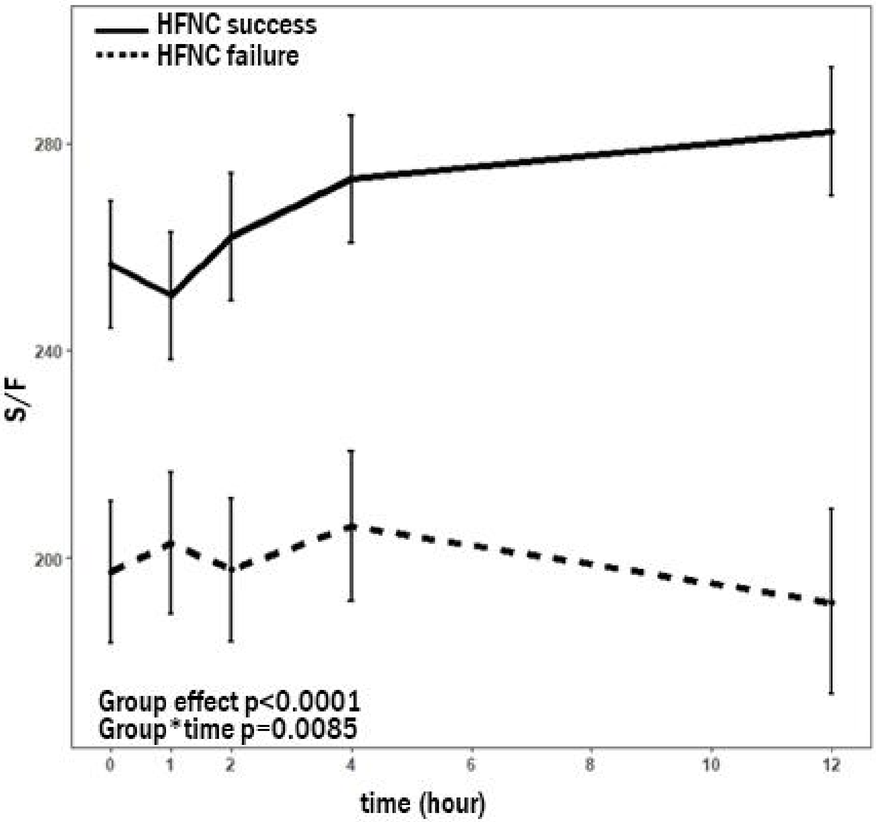

Supplementary Figure 2. Serial mean S/F over time between success and failure groups during HFNC

The mean S/F values were derived from a linear mixed model, with 95% confidence intervals (error bars). The graph shows interaction effects between groups (*P*-value <0.0001) and time *group (*P*-value=0.0085)

S/F, ratio of oxygen saturation and fraction of inspired oxygen (SpO_2)/FiO_2; HFNC, high flow nasal cannula
